# Hospital and Institutional News

**Published:** 1918-03-23

**Authors:** 


### HOSPITAL AND INSTITUTIONAL NEWS.

### ANNUAL MEETINGS AS MONEY-SPINNERS.

WE have maintained for years that many voluntary hospitals lose much each year by not organising the annual meeting and preparing an agenda, full of interest, with good speakers. Each such meeting, properly worked, can produce at trifling expense, from £1,000 to £5,000, according to the size and importance of the institution. Why is this source of revenue not worked by every voluntary hospital? is a question asked by large givers and enthusiastic supporters of the voluntary system throughout the nation. Those institutions which fail to utilise this opportunity reveal themselves as lacking vigour both in the committee and the secretariat. Omitting the largest hospitals, three instances may be quoted as evidence of the precise accuracy of the views just advanced. The "Dreadnought" Seamen's Hospital, which began the system more than thirty years ago and raised £1,500 at its annual meeting, has continued the policy and this year (see p. 531) the results were a bumper. The Poplar Hospital for Accidents, since the dinner was made impossible by the war, has substituted energetic organisation at the annual meeting. This has resulted in £4,013 being raised at the 1914 meeting, £6,814 at that of 1915, £6,131 at that of 1916, and £7,125 at that of 1917. In the provinces the Royal Infirmary, Leicester, is a notable example amongst others of similar results in war-time from energetic action at the centre. The meeting of the Seamen's Hospital Society at the Prince's Restaurant on March 14 constitutes a record. The number of people attending was so great that they more than filled the largest hall, and an overflow meeting had to be held, at which 300 at least were present. Sir Perceval Nairne, the Chairman, Captain Clarke, the Deputy-Chairman, and Mr. Pietro Michelli, the Secretary, are a trio who collectively represent exceptional brains and splendid energy. The same may be said of the managers of the two other institutions we have instanced out of several that might be mentioned. Every voluntary hospital needs and should secure equal power at the centre, when equivalent results would be possible. Example and success are contagious. May they prove to be eye-openers and awakeners throughout the whole voluntary hospital system, wherever the vitalising of the annual meeting and its vigorous conduct are yet to seek.

### BRAIN-WORKERS AND THEIR FOOD.

THE brain-worker, who in these strenuous times labours often longer hours, continuously, with the full exercise of his best powers, than probably any other workers in Great Britain, is usually and necessarily one who eats with discretion and avoids heavy and numerous meals. If he is over middle-age and habitually full of energy it is essential for him, if he is to continue his work for the nation, that he should have the food which long and continuous experience demonstrates to be best and most productive of his full energy. We think it essential to state these facts in view of Lord Rhondda's declaration to the contrary, if he is correctly reported in the Press, as he undoubtedly has been. It is only due in these circumstances to the brains of the nation that Lord Rhondda should publish the names of the experts, if they be experts, whose opinion is described as "scientific and unanimous," who have declared that a man does not need any more food because he works with his brain than he would need if he were not working. Do the scientists in question live on air, or do they over-feed, or what is the explanation of this utterance? In any case the declaration misses the point, for it is not the quantity but the quality and kind of food which are important in this connection. The lengthened period during which brain-workers are kept, sometimes, necessarily and continuously at work, in existing circumstances, may render it essential that they should have a square meal, after the day's work is completed, before entering on the new work which may devolve on them during the night, for example. It is not only the brain-worker but the printer and every intelligent worker who has to do night shifts at the end of a day's work that require this special provision in regard to food. As the decision of Lord Rhondda stands at the moment it amounts to this: that the directing mind, the administrative mind, the productive mind—and where will the nation get to without these?—are to be prevented, or some of them, by rationing, from obtaining the essential food, which may be small in amount, but without which their health and productive powers must suffer, as experience has shown, and may now demonstrate, to the nation's loss.

### A NEW FINANCIAL POLICY AT CARDIFF.

COLONEL BRUCE VAUGHAN was happily sufficiently recovered to preside last week at the monthly meeting of King Edward VII.'s Hospital, Cardiff. As usual, he was full of matter, and put forward not only the needs of the time, but a policy by which these might be met. To complete the War Memorial, he said, £80,000 was required. Apart from two generous private promises, he went on to explain that the future income of the hospital must rise to £40,000. He then pointed out that the alternative to perennial appeals, which have, by the way, been very successful, was a comprehensive scheme in which contributions from all classes should be organised as a voluntary rate. The Lord Mayor, he added, and the presidents of all the principal bodies in the port of Cardiff and the county of Glamorgan were interesting themselves in the scheme. A penny a week from the workmen and an addition of one-fourth of this from employers, together with annual subscriptions from the well-to-do, and the waiting list could be abolished. We are glad to know that this comprehensive scheme for maintaining the necessarily large annual income is likely to be realised shortly. It will be remembered that hitherto the very appeals to the press have risen above the level of casual cries for assistance. A publicity campaign ably conducted has been carried out, and one of the fruits of that campaign is now seen, not in the immediate sums subscribed under its influence, but in the readiness of all classes to adopt the new policy of sectional subscriptions.

### DEMOCRACY AND MANAGEMENT.

The growth of the representation of the working classes on the committees of the Bristol hospitals is beginning to settle down steadily. The early days of doubts and fiction, assertion on the one hand and suspicion on the other, are being replaced by a spirit of co-operation advantageous alike to the workmen and the institutions. The Lord Mayor, at the recent annual meeting of Bristol General Hospital, hinted that a scheme was in hand which would ensure further co-operation, more generous gifts on the one side and fuller representation on the other. With the experience of the Midland and Northern hospitals to guide them, it should be possible to avoid the difficulties which hampered the work in other places, difficulties of which unmanageable committees of enormous size were perhaps the chief. The machinery of democratic control has too long been confused with the reality, and so long as any section is really able to make its voice heard in hospital management the reality of democratic control is assured. The farce of the House of Commons, a committee of 670 or so, should warn us all that mere numbers guarantee nothing but party voting, waste of time, and endless wire-pulling.

### THE END OF INACTION AT DEVONPORT.

At last, after a long period of ineffective struggle against debt, the Royal Albert Hospital, Devonport, has fixed July 1 as the date on which it will consider closing its doors unless £12,500 shall have been subscribed in the interval. This announcement is simply the inevitable result of a half-hearted policy of drift, and is more significant as a proof of the end to which this leads than as in itself an alarming statement. It is, we know, always difficult to start an uphill fight, but the fact remains that Devonport can do what other towns have done, especially in these days, when the public is more awake than ever to the needs of hospitals. It would be better if a plan of campaign took the place of the paragraph on closing, to which we have referred. For such a plan implies a positive effort, while the bare mention of closing implies that the possibility of failure is accepted by the Hospital's board. The organisation of sectional subscriptions from every separate class of the community which the hospital serves has proved highly successful in North Staffordshire. Mr. Rea has shown how a newspaper campaign can be conducted at Cardiff. With these examples before them, the board of the Royal Albert Hospital, Devonport, have no excuse for not finding methods to hand for raising much more than the modest sum for which it asks.

### BEGGING IN THE WARDS.

Those who misunderstand the principles which lie at the root of the voluntary system often fall into the error of supposing that the need for money justifies any expedient by which it may be raised. In the main the follies which result are attempted by foolish persons unconnected with the hospitals, but occasionally a hospital is itself to blame. This seems to be the case in connection with the Herefordshire General Hospital, where a member of the board, Mr. G. W. Bellers, has objected to the practice "of sending children round to patients' visitors asking them to contribute money to a box." The board once agreed with Colonel Hewat that the practice should not be allowed, and the House Committee has been looking into the matter. It is suggested that the practice occurred at Christmas, when the children may have taken the collecting box in their ward and rattled it in front of their visitors. If they did we hope it is not too late to discover who suggested the notion to them, so that the person in question may be brought to her senses. Probably the whole thing arose, behind the board's back, on the initiative of some foolish individual, but nothing more opposed to financial results, can be imagined.

### THE WAITING LIST AT NOTTINGHAM.

The necessity for an early extension of the General Hospital, Nottingham, is apparent from the fact that the waiting list to-day numbers over 1,000. This, bluntly, is an intolerable state of affairs. The long waiting list has been till lately a tradition, an evil, that is to say, all the worse from having become accepted as a matter of course. It is evils like these, rather than calamities, which are so hard to eradicate. And yet it is not too much to say that the voluntary system has been more imperilled from this defect than from any other. Still the public conscience can be awakened by persistent exposure of the evil, and, with the encouraging example of Cardiff before him, Mr. F. Acton should be able to lead a reaction in Nottingham which will at least provide the money for the new buildings which are required.

### THE DEVELOPMENT OF WORKPEOPLE'S CONTRIBUTIONS.

"Each year since the foundation of the Society in 1903 we have been happy to announce an augmented income." These words are quoted from the report of the Leicester and County Saturday Hospital Society, which was presented to the delegates and members at the annual meeting held at the Leicester Royal Infirmary. Mr. W. J. Gipson Clarke, the newly elected president of the Society, in the place of the late Sir Edward Wood, laid great stress upon the happiness of the co-operation between the Infirmary and the Society, and intimated that the total sum collected during the year amounted to £17,442, an increase of £456 on the preceding year. The report paid eloquent tribute to the fostering care and untiring interest of Sir Edward Wood, the founder and first president, and it was decided to invite each of the members to contribute to a memorial fund, part to be given to the Sir Edward Wood Wing at the Infirmary, and the balance to the provision of medallions of Sir Edward Wood in the two convalescent homes of the Society. The Desford Hall Home for men had received 566 soldier patients during the year, an increase of 227, and the Swithland Home for male and female civilian cases had received 654 patients, an increase of twelve. The after effects of the war were beginning to appear, and the Society had agreed to accommodate discharged wounded soldiers needing convalescent treatment when beds were available. The work of Mr. H. H. Woolley, J.P., the zealous secretary and organiser, was feelingly recognised, and on behalf of the board of the Infirmary, Mr. T. Fielding Johnson, for the chairman, warmly acknowledged the immense work which the Society had accomplished, and its great influence in the maintenance of the work of the Infirmary. He might even have added that its example has done much to foster the organisation of working-class subscriptions in other centres, and that when such efforts are made the example of the Leicester workpeople is usually held up as an inspiring model.

### THE QUEEN AND THE CHELSEA HOSPITAL FOR WOMEN.

H.M. the Queen, accompanied by Princess Mary, was present at the Palladium on the 15th inst., the occasion being the matinée performance in aid of the funds of the new Chelsea Hospital for Women. In the unavoidable absence of her husband, who is President of the Hospital, Lady Londonderry made a short speech and thanked all those who had combined to make the function so successful, the speaker's mention of Her Majesty's kindness being received with hearty applause by the large audience. The resulting amount (£3,000 to date) will substantially reduce the debt on the new buildings.

### THREE GIFTS TO QUEEN'S HOSPITAL, BIRMINGHAM.

Through the generosity of Mr. and Mrs. Lionel Spiers, the Queen's Hospital, Birmingham, has opened a department for electrical and mechanical treatment, the installation for which has been given by them as a memorial to their son, Lieut. A. L. Spiers, who was killed in action last September. The new department, however, is cramped through lack of space in the temporary rooms in which the department is working. The employés of the Wolsey Motor Company have given to the hospital a motor ambulance to convey wounded soldiers, and Mr. W. H. Abbott, who drives the car, has paid all the expenses of its upkeep. Another gift is that of an open-air shelter, presented by Messrs. J. Turton and Company. These three gifts have enabled the hospital to increase the variety and usefulness of its work, which still maintains 163 beds for civilians, a decrease of 13 since the beginning of the war.

### THE NEW PRESIDENT OF THE ROYAL LANCASTER INFIRMARY.

Mr. Williams Matthews Duncan, who has just been appointed President of the Royal Lancaster Infirmary, is a Scotsman, who was born in Charlotte Square, Edinburgh, in 1860, and is the son of a distinguished physician, Dr. James Matthews Duncan, assistant to Sir James Simpson, the discoverer of chloroform. The story goes that Dr. Duncan was probably the first person to be put under chloroform. He was also lecturer and physician-in-charge of the midwifery wards at Edinburgh Infirmary. He afterwards went to St. Bartholomew's Hospital, London, where he had a distinguished career. He was LL.D. and F.R.S., and was the only obstetrician ever elected President of a Royal College of Physicians. Mr. W. M. Duncan, the new President, was educated at Fettes and Trinity College, Cambridge, and his connection with Lancaster began with his friend Mr. Edgar Storey in 1874. Mr. Duncan joined the Rembrandt Intaglio Printing Company in 1894, before it became a limited company, and has lived in Lancaster since then. He is a keen sportsman, was first captain of the Lancester Golf Club, and also first captain of the Lancester Rifle Club. He is a special constable, and is on the committee of the Royal Lancaster Cadet Corps. He has been a member of the committee of the Royal Lancaster Infirmary since 1904.

### MR. ROBEY'S PRESENTATION.

The recent presentation to Mr. George Robey for having raised a sum of £50,000 for the benefit of war charities is interesting in more senses than one. Not only is it a tribute to Mr. Robey's unfailing popularity, and to the eagerness with which the theatrical profession has always offered its services in the cause of charity, but it ended in the happy decision of Mr. Robey to present the cheque for £250, which accompanied the presentation of an address with a silver service, to the Music Hall Benevolent Fund. The choice was left to him as to the destination of the cheque, and it is pleasant for hospital men to note that his work for them has resulted in such a gift to a theatrical charity. Hospitals have been so often helped by the Stage that they will appreciate the benefit now coming to the Music Hall Benevolent Fund from the fund raised in recognition of Mr. Robey's services to war charities.

### SECTIONAL SUBSCRIPTIONS IN CATHEDRAL TOWNS.

The St. Albans and Mid-Herts Cottage Hospital has suffered a loss in the resignation of its honorary secretary, Mrs. Leighton, who has held the post for about ten years. It was she who instituted the Ladies' Collecting Committee, which has done much year after year to organise subscriptions and donations in the city and the neighbourhood. Mrs. Leighton has been able to devote her energies to work of this character, since the hospital possesses also a secretary, Mr. W. G. Marshall, on whom the necessary daily duties fall. It is interesting to note that St. Albans, which has not the reputation of an industrial centre, but rather suggests the quiet of a cathedral town, assists its hospital by weekly collections from the firms and employees in the city and district. It will be seen how these collections, coupled with those organised by Mrs. Leighton, prove once again that the way to organise sectional subscriptions it to form a separate scheme suited to every class served by a hospital.

### HAHNEMANN AND PUBLIC CONFIDENCE.

In dealing with the position of the Hahnemann Hospital, Liverpool, in regard to the College of Nursing we recorded its receipt of a decreased grant from the Hospital Saturday and Sunday Funds. Mr. R. M. Ainslie and Mr. H. S. Coghill, the respective secretaries of the two Funds, have reminded the hospital's chairman, Mr. Carlton Stitt, that the reduction was made because the income of the Hahnemann Hospital for 1916 exceeded its expenditure by £506, " and the treasurer was able to carry forward a credit balance of £805 after investing £300." Reduced grants were also made to two other local institutions whose position did not seem to warrant, in the view of the Fund, the extent of its previous assistance. The position should be made clear, we think, since hospitals named after Hahnemann are apt to suppose that they are liable to preferentially less generous treatment than others.

### THE PRINCE OF WALES' INSPIRATION.

Even the war failed to move the people of Cardiff to complete the Jubilee Fund in connection with the Royal Hamadryad Seamen's Hospital. But in consequence of the Prince of Wales' visit the total was quickly raised to £25,000, and a week later a further £10,000 was subscribed. The patients include a large number of Royal Naval Reserve men and many seamen from torpedoed vessels. The reopening of the Jubilee Fund is one of the happy legacies of the Prince of Wales' visit, God bless him.

### SAILORS' AND SOLDIERS' DEPENDANTS IN RATE-AIDED INSTITUTIONS.

In The Hospital of October 25 reference was made to the action of the Army Council in asking Boards of Guardians to notify when dependants of sailors and soldiers were admitted to rate-aided institutions. This had been done in some instances, when it was found that the Army Council at once stopped the separation allowances, and that in many instances cases of hardship ensued. Many Guardians thereupon declined to give the Army Council any further information. The Local Government Board has now notified the Guardians that the War Cabinet has decided to modify the order. In future the separation allowance will be continued as usual at the old rate when a dependant, other than the wife or unmarried wife of a sailor or soldier, is admitted to a rate-aided institution, but in the cases of the wives and unmarried wives the allowances will be stopped, and arrangements made with the Local War Pensions Committee for an allowance in lieu of, but at the same rate as, the separation allowance whilst the women are in the institution. This will also apply to the widows of sailors and soldiers. It is doubtful if this modified proposal will meet with the approval of the Boards of Guardians who previously objected to the old order, as it may lead to undue delay between the time the allowances are stopped and before the Local Pensions Committees take action. This is the chief objection to the old order over again. Will the Guardians be any more likely to comply with it?

### TETANUS GERMS IN CATGUT.

Dr. Wynn Westcott, Coroner for the North-Eastern District, at an inquest on a four-year-old girl, a soldier's daughter, who died in the Metropolitan Hospital from lockjaw, remarked that if persons were operated on, as this child had been, for hernia they had to take a considerable risk. It was, however, very unusual for a child to contract lockjaw in this way, and he had not heard of such a case in London for many years. There were soldiers in the institution, and, of course, they might have the germs in their clothing. There had been tetanus germs found in gelatine and catgut used for tying up wounds. The house surgeon at the hospital said they had separate operating-rooms for soldiers, and no case of the kind was known amongst them. The instruments in use were being examined, and in every way they were trying to find out how the germs had been conveyed. The jury returned a verdict of " Death by misadventure."

### WAR PRICES AT RHYL.

The difficulties of the time and the abnormal cost of living have pressed heavily on the Royal Alexandra Children's Hospital and Convalescent Home, Rhyl; it has to report a deficit of over £400 on the year's expenditure of £7,527. The subscriptions (£1,330) showed a small increase, and the private nurses, notwithstanding that on an average ten of the nurses have been away working in military hospitals, again did well, their earnings amounting to £2,377, an increase of £214. A satisfactory feature of the year's record is that, thanks to generous gifts of game, vegetables, and fruit, the average weekly cost of food per head for patients and staff was kept at the comparatively low figure of 6s. 7d. The number of patients treated during the year was 666. It is interesting to note that five of the hospital's nurses have been awarded the Médaille d'Honneur for zeal and devotion shown in nursing wounded men in France.

### THROUGH THE EYES OF A CHILD.

To the recent records of long service which we published in respect of two northern hospitals we should like to add one more, which concerns, however, a single individual. Mr. Crewdson, one of the auditors of the Southport Convalescent Hospital, is able to boast that his associations with it cover nearly sixty years, and extend to the time when as a child he knew it as a mysterious place whose precise function he was not able to divine. To retain that sense of mystery is the proof of a long and vivid memory, which has made the hospital live as a central fact in the mind of one who recalls its early effect upon his imagination.

### A DISPENSER'S RECORD OF SERVICE.

Lengthy associations with institutions, like that recorded of an auditor in the above note, are not the same as actual periods of daily work. An example of the latter is given by Mr. Charles T. Carter, who has held for forty-eight years the post of dispenser to the Dorset County Hospital.

